# Evaluating the technetium-99 m pertechnetate flow protocol for Graves’ disease: methodological insights: a case series

**DOI:** 10.1186/s13256-025-05365-1

**Published:** 2025-07-01

**Authors:** Jaber Abdulwahab Asiri, Rami Abdullah Alghamdi, Ahmed Ali AlRizqi, Shahad Khalaf Alrasheed, Abdulaziz Alaythan, Bander Mukhlef Alshammri, Mohammed Abdullah Alshehri, Mohammed Hadi Alarjani, Zyad Alshehri, Nahed Nasser ALFawaz, Rayan Saad Albalawi

**Affiliations:** King Saud Medical City, Ulaishah, Riyadh, 12746 Kingdom of Saudi Arabia

**Keywords:** Graves’ disease, Dynamic imaging, Thyroid scan scintigraphy

## Abstract

**Background:**

Graves’ disease is the most prevalent cause of hyperthyroidism globally, with technetium-99m pertechnetate widely utilized in its diagnosis. Dynamic scintigraphy using technetium-99m pertechnetate has shown promise for early detection of Graves’ disease, particularly in subclinical presentations, but lacks standardized evaluation. This study aims to explore the utility of a dynamic technetium-99m pertechnetate scintigraphy protocol in hyperthyroidism, focusing on its diagnostic advantages and potential clinical applications.

**Case presentation:**

The cases described involved adult male and female patients aged 34–40 years of Saudi Middle Eastern ethnicity.

**Conclusions:**

Dynamic flow imaging using technetium-99m pertechnetate represents a promising diagnostic approach for Graves’ disease, particularly in challenging subclinical cases. This protocol has the potential to refine diagnostic accuracy and guide treatment decisions. Further validation through larger studies is warranted to establish its clinical utility.

## Introduction

Hyperthyroidism occurs when the thyroid gland produces an excessive amount of hormones. It can be categorized into two categories: subclinical or overt. Subclinical hyperthyroidism is characterized by suppressed thyroid-stimulating hormone (TSH) with normal thyroxine (T4) and triiodothyronine (T3) levels. In contrast, overt hyperthyroidism is defined by suppressed TSH and elevated T3 and T4 levels [1].

The three primary causes of hyperthyroidism are Graves’ disease, toxic adenoma, and toxic multinodular goiter. Less common etiologies include ectopic thyroid tissue in struma ovarii and iodine-induced hyperthyroidism [1]. Graves’ disease, the most common cause, accounts for up to 80% of hyperthyroidism cases and affects four out of five patients with this condition. It predominantly affects individuals aged 20–50 years, with lifetime risks of 0.5% in men and 3% in women [2, 3]. Beyond the thyroid gland, Graves’ disease can impact other organs, such as the eyes and the skin, making its diagnosis critical [3].

Diagnostic evaluation typically begins with laboratory tests, such as TSH, T3, and T4. A low TSH with elevated T3 or T4 indicates overt hyperthyroidism, while normal T3 and T4 levels suggest subclinical hyperthyroidism. Additional diagnostic parameters include the T3/T4 or FT3/FT4 ratios, where values exceeding 20 (ng/mcg) or 0.3, respectively, support a diagnosis of Graves’ disease [3]. Obtaining a detailed patient history is essential, particularly when family history, diffuse thyroid enlargement, or orbitopathy suggests Graves’ disease [3].

Imaging plays a key role in diagnosis. Ultrasound with Doppler assesses thyroid hypervascularity, while radioactive iodine uptake scans (I-123 or I-131) confirm diagnoses. Graves’ disease presents as high, diffuse uptake on iodine uptake scans, whereas toxic nodules and multinodular goiters exhibit focal or heterogeneous uptake patterns [3]. Tc-99m pertechnetate (99mTcO_4_^−^) is another widely used imaging modality for Graves’ disease owing to its availability, lower radiation dose, and cost-effectiveness compared with iodine isotopes. Tc-99m uptake values between 0.5% and 4% are considered normal, with elevated values indicating hyperthyroidism [4].

The standard protocol for 99mTcO_4_^−^ involves intravenous (IV) injection followed by static imaging after 15–20 minutes [5]. Hyperthyroidism encompasses various types, including multinodular toxic goiter (MTG), Morbus Graves–Basedow (MGB), and Plummer disease [6, 7]. Despite this diversity, most hospitals and clinical sites rely on a standard Technetium-99m thyroid uptake (TcTU) protocol. A persistent issue observed in practice is the discordance in some cases, where patients with clinical signs of hyperthyroidism and supportive laboratory tests exhibit negative Technetium-99m thyroid uptake (TcTU) findings that do not align with their symptoms or overall clinical presentation.

## Experimental design and equipment

Dynamic 99mTcO_4_^−^ pertechnetate scintigraphy has been previously used in research and case reports to detect Graves’ disease at an early stage. At King Saud Medical City, Riyadh, Saudi Arabia, this method was implemented to perform dynamic 99mTcO_4_^−^ scintigraphy in patients with hyperthyroidism. This case series highlights the value of combining flow and dynamic techniques with the regular protocol for 99mTcO_4_^−^ scintigraphy to enhance the evaluation of hyperthyroidism. In addition, findings were correlated with the thyroid-stimulating hormone receptor antibodies (TRAB) laboratory test to provide a comprehensive diagnostic approach.

To improve the diagnostic sensitivity for Graves’ disease, four parameters were introduced: early blood flow (EBF), perfusion index (PI), and uptake indices 1 and 2 (UI1 and UI2). EBF was calculated by drawing regions of interest (ROI) around the thyroid gland during the first minute of imaging to generate a time–activity curve. The duration of early blood flow was determined based on the curve. PI was calculated as the ratio of counts at the start of early blood flow divided by counts at its end. UI1 and UI2 were calculated as the ratios of specific counts during dynamic imaging phases, ensuring a robust assessment of thyroid activity.

The normal reference ranges for these four parameters were established using mean ± 2 SD from prior studies [5]. The estimated normal duration of EBF is 13–15 seconds, PI values range from 0.143 to 0.298, UI1 ranges from 1.1 to 2.5, and UI2 ranges from 0.9 to 1.0. These reference values were applied to validate the diagnostic reliability of the method and standardize assessments.

## Detailed procedure

Before the scintigraphy procedure, the patient underwent a thyroid ultrasound and endocrine laboratory tests (TSH, T3, and T4). Patients were instructed to discontinue thyroid medication 5–7 days before the scan and avoid seafood or sodium-rich food during this period, in accordance with the nuclear medicine department’s protocol at King Saud Medical City. In addition, patients were required to avoid undergoing any computed tomography (CT) with contrast for 6–8 weeks before thyroid scan scintigraphy.

On the day of the procedure, an IV cannula was inserted, and the patient was positioned supine, headfirst, under the GE Discovery NM/CT 670 scanner. A pre- and postsyringe scan was performed using the gamma camera to calculate Technetium-99m thyroid uptake (TcTU) using the formula

$$\% {\text{ uptake}} = \left[ {\left( {{\text{thyroid count}} - {\text{background count}}} \right)/\left( {{\text{pre - injection syringe count}} - {\text{post injection syringe count}}} \right)} \right] \times {1}00\%.$$

The patient received an intravenous injection of 5 mCi 99mTcO_4_^−^ pertechnetate under gamma camera guidance. Imaging parameters for the flow technique included a 1-minute acquisition with 20 frames, each lasting 3 seconds, at a zoom of 1.45. A 20-minute dynamic acquisition was performed with 20 frames covering 60 seconds, followed by 3-minute anterior static imaging at a zoom of 3.

## Case presentations

### Case 1

A 37-year-old Saudi female was referred to the endocrinology department for assessment of hyperthyroidism.

**Fig. 1:**
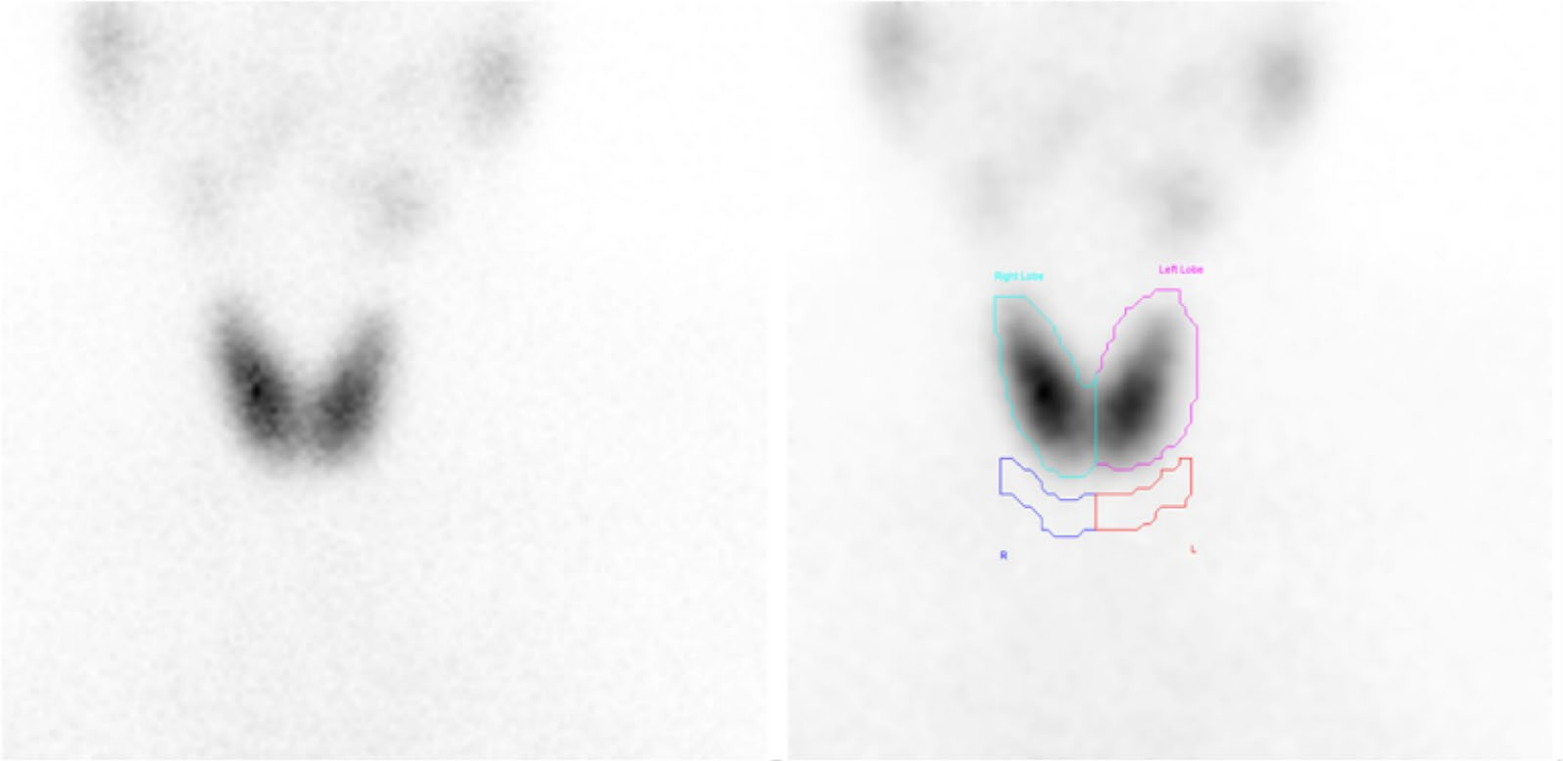
Normal thyroid uptake at 3.4% for case 1. The thyroid gland is in its normal position, demonstrating homogeneous tracer distribution with mild increased uptake. No cold nodules are observed

As illustrated in Fig. 1, static thyroid scintigraphy showed normal uptake at 3.4%, with uniform tracer distribution and no evidence of cold nodules.

**Fig. 2:**
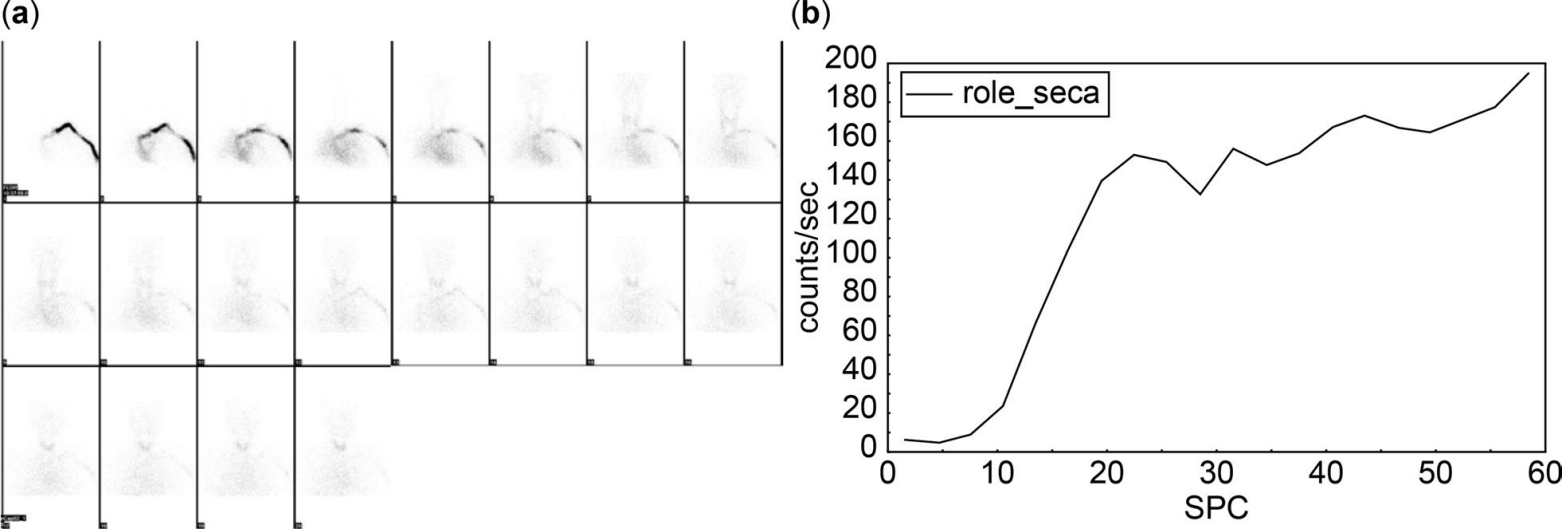
Increased thyroid vascularity and early blood flow parameters for case 1. **a** Mildly increased thyroid vascularity is observed, with the thyroid gland appearing in frame 7 at 21 seconds postinjection. **b** Early blood flow begins at 10.5 seconds and ends at 19.5 seconds, lasting 9 seconds. Perfusion index is calculated at 0.12, while the uptake indices indicate that uptake index 1 and uptake index 2 are 0.75 and 0.8, respectively

Dynamic imaging indicated increased thyroid vascularity and early blood flow, with the thyroid gland first visible in frame 7, occurring 21 seconds after tracer injection. The early blood flow phase started at 10.5 seconds and ended at 19.5 seconds, lasting a total of 9 seconds. The perfusion index (PI) was measured at 0.12. Uptake indices were recorded as UI1 of 0.75 and UI2 of 0.8, as shown in Fig. 2.

### Case 2

**Fig. 3:**
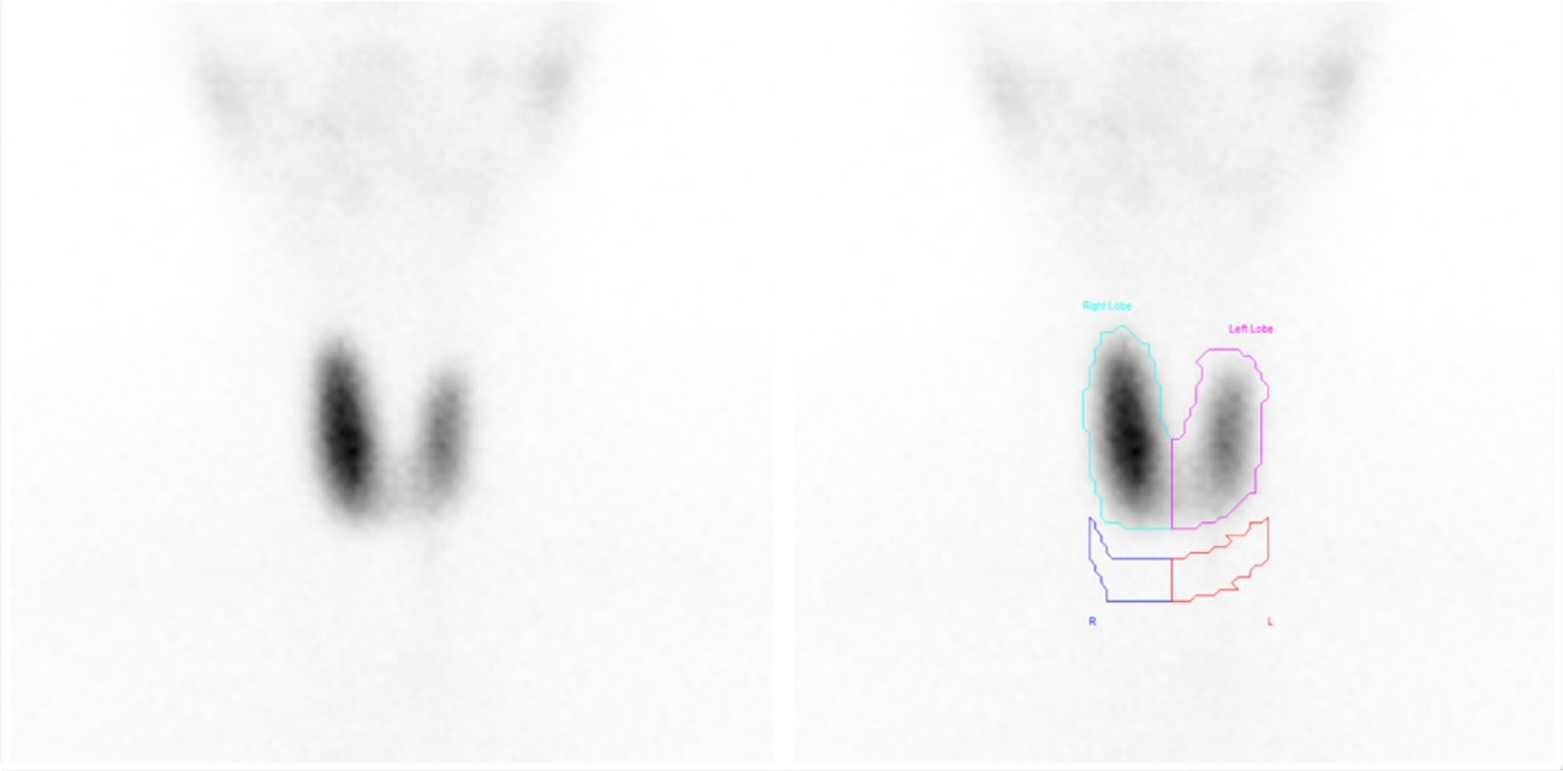
Enlarged right thyroid lobe with increased tracer uptake in case 2. The thyroid gland demonstrates an enlarged right lobe compared with the left, with homogeneous tracer distribution in both lobes. Tracer uptake is more prominent in the right lobe than in the left, with a total thyroid uptake of 6%

**Fig. 4:**
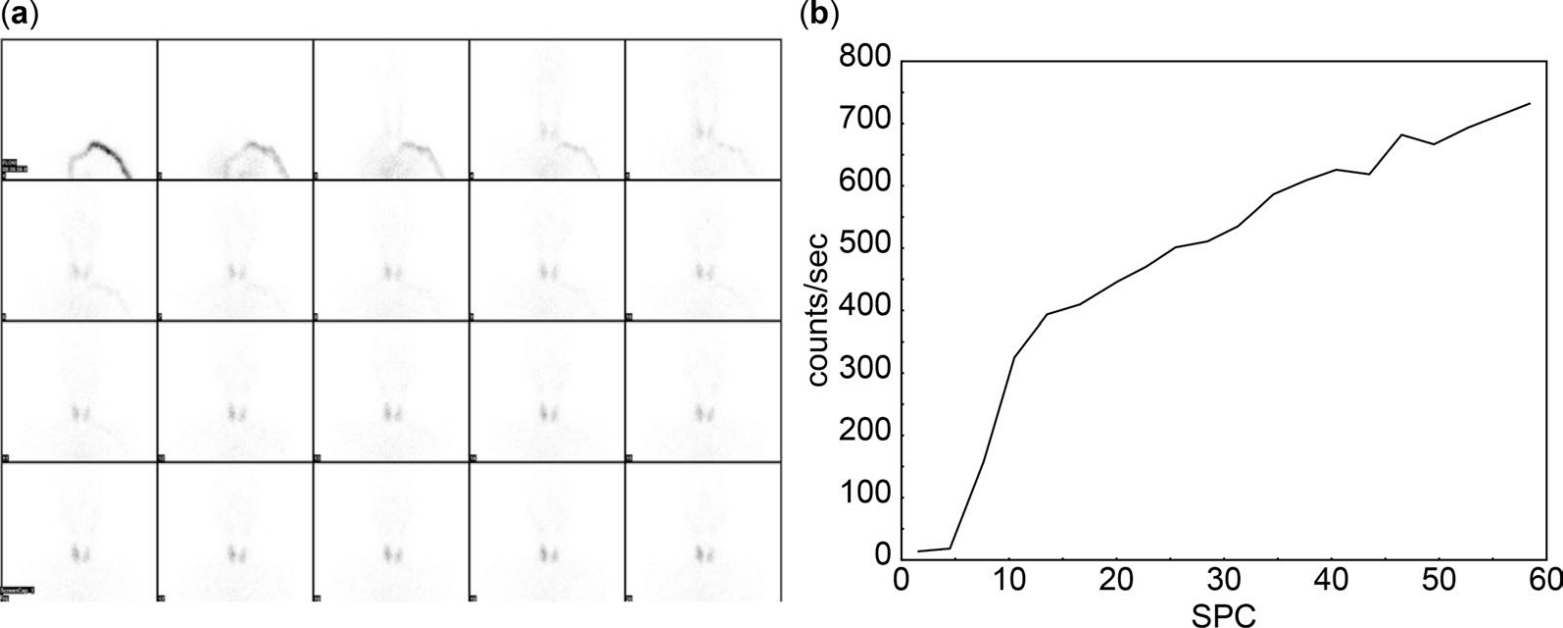
Hyperemia and early blood flow parameters in thyroid lobes in case 2. **a** Flow images reveal hyperemia in both thyroid lobes, with initial visualization occurring in frames 3–4 at 12 seconds postinjection. **b** Early blood flow starts at 4.5 seconds and ends at 10.5 seconds, lasting 6 seconds. Perfusion index is 0.11, while uptake indices, uptake index 1 and uptake index 2, are 0.5 and 0.73, respectively

A 34-year-old Saudi female presented with a 1-year history of anxiety, insomnia, hypertension, palpitations, protruding eyes, and significant weight loss. Laboratory results showed a mildly decreased TSH of 0.05, with normal T3 and T4 levels. The thyroid uptake was elevated at 6.2% (reference: 0.4–4%) (Fig. 3). Imaging findings, including EBF, PI, UI1, and UI2, supported the diagnosis of Graves’ disease (Fig. 4). Post-scan thyroid-stimulating hormone receptor antibodies (TRAB) results were elevated at 5 IU/L, confirming the diagnosis.

### Case 3

**Fig. 5:**
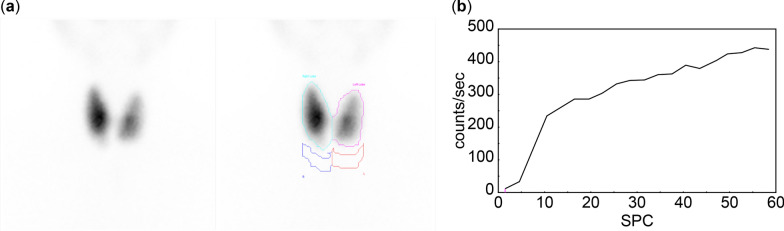
Enlarged thyroid gland with increased isotope uptake and early blood flow parameters in case 3. **a** The thyroid gland is enlarged, showing homogeneous isotope uptake in both lobes, with a total thyroid uptake of 22%. **b** Early blood flow begins at 4.5 seconds and ends at 10.5 seconds, lasting 6 seconds. Perfusion index is 0.05, while uptake indices, uptake index 1 and uptake index 2, are 0.38 and 0.8, respectively

A 40-year-old Saudi male patient with diabetes and a 2-year history of hyperthyroidism presented with persistently abnormal thyroid laboratory results (TSH: 0.0083 uIU/mL, FT3: 4.88 pmol/L, FT4: 10.09 pmol/L) despite escalating carbimazole doses up to 60 mg. Thyroid uptake was markedly elevated at 22%, and flow imaging indicated hypervascularity (Fig. 5). Graves’ disease was confirmed, and the patient was subsequently treated with a low dose of I-131.

As summarized in Table 1, the values of EBF, PI, UI1, and UI2 across the three cases illustrate the variability in thyroid perfusion and uptake, supporting diagnostic distinctions even when laboratory results are inconclusive.

**Table 1:** Summary of dynamic imaging parameters across cases

Case	EBF (s)	PI	UI1	UI2
Case 1	9	0.12	0.75	0.80
Case 2	6	0.11	0.50	0.73
Case 3	6	0.05	0.38	0.80

EBF = Early blood flow; PI = Perfusion index; UI1 = Uptake Index 1; UI2 = Uptake Index 2

## Discussion

The assessment of thyroid vascularity with 99mTcO_4_^−^ scintigraphy has been validated in numerous studies, particularly in patients with Graves’ disease, where hyperemia is commonly observed [5]. This study utilized key parameters (EBF, PI, UI1, and UI2) derived from the time–activity curve (TAC). The TAC is generated by drawing regions of interest (ROI) over the thyroid gland during the first minute of flow imaging, enabling precise calculations of these parameters. Normal values for EBF (13–15 seconds), PI (0.143–0.298), UI1 (1.1–2.5), and UI2 (0.9–1.0) were used as reference points [8].

The findings in cases 1, 2, and 3 demonstrate the utility of these parameters in evaluating Graves’ disease. In case 1, despite normal thyroid function test results, the abnormal thyroid-stimulating hormone receptor antibodies (TRAB) value and deviations in PI, UI1, and UI2 confirmed the diagnosis. Case 2 highlighted the abnormal flow imaging findings, including reduced EBF duration (6 seconds) and PI, UI1, and UI2 values below the normal range. Similarly, in case 3, the elevated thyroid uptake (22%) and abnormal parameter values confirmed hyperthyroidism, further validating the diagnostic significance of these measures.

## Conclusion

The results confirm the value of 99mTcO_4_^−^ scintigraphy in diagnosing Graves’ disease, particularly in challenging cases, such as those with subclinical presentations. Incorporating flow imaging and novel diagnostic parameters enhances sensitivity and provides deeper insights into thyroid vascularity. Further studies with larger sample sizes are recommended to validate these findings and explore the utility of these parameters in broader clinical settings.

## Data Availability

Nearly all relevant data are presented in the manuscript.

## Abbreviations

EBF: Early blood flow
PI: Perfusion index
UI1: Uptake index 1
UI2: Uptake index 2
TRAB: Thyroid-stimulating hormone receptor antibodies
TSH: Thyroid-stimulating hormone
FT3: Free triiodothyronine
FT4: Free thyroxine
TcTU: Technetium-99m thyroid uptake
ROI: Region of interest
99mTcO_4_^−^: Technetium-99m pertechnetate
TAC: Time–activity curve
